# Chronic Neurobehavioral Impairments and Decreased Hippocampal Expression of Genes Important for Brain Glucose Utilization in a Mouse Model of Mild TBI

**DOI:** 10.3389/fendo.2020.556380

**Published:** 2020-09-18

**Authors:** Linda M. Huynh, Mark P. Burns, Daniel D. Taub, Marc R. Blackman, June Zhou

**Affiliations:** ^1^Research Service, Washington DC VA Medical Center, Washington, DC, United States; ^2^Department of Neuroscience, Georgetown University School of Medicine, Washington, DC, United States; ^3^Department of Biochemistry and Molecular and Cell Biology, Georgetown University School of Medicine, Washington, DC, United States; ^4^Department of Medicine, George Washington University School of Medicine, Washington, DC, United States; ^5^Department of Medicine, Georgetown University School of Medicine, Washington, DC, United States

**Keywords:** gene expression, glucose metabolism, pyruvate kinases, pyruvate dehydrogenase, chronic TBI, aging, neurobehavior tests

## Abstract

Glucose is an essential cellular fuel for maintaining normal brain functions. Traumatic brain injury (TBI) decreases brain glucose utilization in both human and experimental animals during the acute or subacute phase of TBI. It remains unclear as to how the damages affect brain glucose utilization and its association with persistent neurobehavioral impairments in the chronic phase of mild TBI (mTBI). Accordingly, we compared expression of selected genes important to brain glucose utilization in different brain regions of mice during the chronic phase in mTBI vs. sham operated mice. These genes included hexokinase-1 (HK1), phosphofructokinase (PFK), pyruvate kinase (PK), pyruvate dehydrogenase (PDH), capillary glucose transporter (Glut-1), neuron glucose transporter (Glut-3), astrocyte lactate transpor1 (MCT-1), neuron lactate transporter (MCT-2), lactate receptor (GPR81), and Hexokinase isoform-2 (HK2). Young adult male C57BL/6J mice were brain injured with repetitive closed-head concussions. Morris water maze (MWM), elevated plus maze (EPM), and neurological severity score test (NSS) were performed for evaluation of mice neurobehavioral impairments at 2, 4, and 6 months post mTBI. Two days after completion of the last behavioral test, the frontal cortex, hippocampus, brainstem, hypothalamus, and cerebellum were collected for gene expression measurements. The expression of the mRNAs encoding PK, and PDH, two critical enzymes in glucose metabolism, was decreased at all-time points only in the hippocampus, but was unchanged in the brainstem, hypothalamus, and cortex in mTBI mice. mTBI mice also exhibited the following behavioral alterations: (1) decreased spatial learning and memory 2, 4, and 6 months after the injury, (2) increased proportion of time spent on open vs. closed arms determined by EPM, and (3) accelerated reduction in motor activity observed at 4 months, two months earlier than observed in the sham group, during the EPM testing. There were no significant differences in NSS between injury and sham groups at any of the three time points. Thus, mTBI in male mice led to persistent decreased hippocampal expression of mRNAs that encode critical glucose utilization related enzymes in association with long-term impairments in selected neurobehavioral outcomes.

## Introduction

Traumatic brain injury (TBI) is defined as a sudden injury to the brain caused by an external physical force applied to the head, such as a blow, bump or jolt (1). In 2017, there were more than 1 million TBI related inpatient stays and emergency department visits in United States (2). Most TBI injuries are classified as mild, rather than moderate or severe (2, 3). The clinical definition of mild TBI (mTBI) includes one or more of the following: “confusion or disorientation, loss of consciousness for 30 min or less, post traumatic amnesia for <24 h, and/or other transient neurological abnormalities such as focal signs, seizures, and intracranial lesion not requiring surgery; and Glasgow Coma Scale score of 13–15 after 30 min post-injury or later upon presentation for healthcare” (4, 5), Concussion is the most common cause of mTBI (2, 3).

Mild traumatic brain injury (mTBI) can lead to long-term and progressive neurological consequences, resulting from a cascade of cellular and molecular responses triggered by primary brain injury (6–8). The cascade of such responses can easily propagate to and damage the other brain tissues that are not on the primary injury site. Amelioration of functional outcomes following mTBI relies on mechanistic understanding of these cellular and molecular responses, especially during the chronic phase of mTBI.

Brain cells use glucose and its related intermediate metabolites as energetic substrates to support their functions. Brain glucose supply and utilization are also closely related to many neurobehavioral functions, such as learning and memory (9–11). Under most normal and pathological conditions, brain glucose utilization is well preserved by complex mechanisms, regardless of peripheral glucose fluctuations. However, TBI consistently triggers transient increases and prolonged decreases in brain glucose utilization, as assessed by brain imaging in both TBI patients and experimental animals (12–17). Regardless of this consistent observation, how mTBI impacts brain glucose utilization at the molecular level is poorly understood, especially during the chronic phase of mTBI.

**Figure 1:**
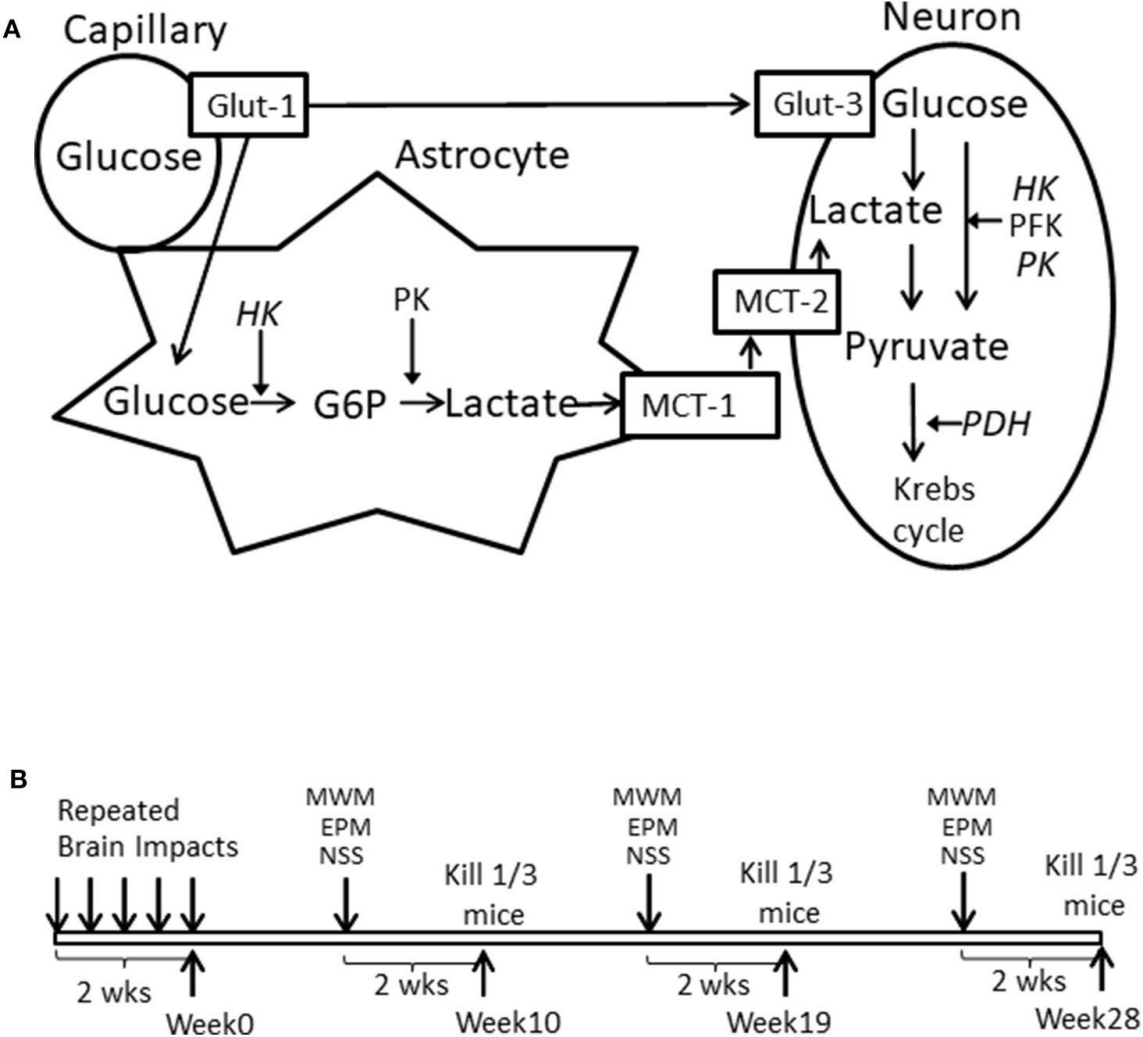
**(A)** Schematized brain glucose utilization among capillaries, astrocytes and neurons. HK, Hexokinase; PFK, phosphofructokinase; PK, pyruvate kinase. PDH, Pyruvate dehydrogenase; Glut-1, Capillary Glucose transporters 1; Glut-3, Neuronal glucose transport 3; MCT-1, Astrocyte monocaboxylate (lactate) transporters 1; MCT-2, neuronal monocaboxylate (lactate) transporters 2. **(B)** Illustration of experimental schedule.

Brain glucose utilization requires glucose transport and metabolism, as schematized in Figure 1A. Glucose transporters (Glut-1 and Glut-3) transport glucose from capillaries to astrocytes and/or neurons (18, 19). Hexokinase (HK1), phosphofructokinase (PFK), pyruvate kinase (PK), and pyruvate dehydrogenase (PDH) are four important rate-limiting enzymes in glucose metabolism. Neurons can also use lactate as an energy substrate to produce ATP (20, 21). Lactate can be produced by glycogenolysis in astrocytes and transported to neurons via monocaboxylate transporters (MCT-1 and MCT-2) (19, 22, 23). HK2 (an isoform of Hexokinase), and GPR81 (lactate receptor) are also important in brain glucose metabolism, especially in the injured brain. HK2 expression has been identified in the outer membrane of mitochondria in most brain areas and is related to abnormal glycolysis during hypoxia and apoptosis (24). Lactate-activated GPR81 affects several biological pathways in the injured brain, including brain glucose metabolism (25–27). The expression of these enzymes and transporters in various brain tissues is vulnerable to a cascade of cellular and molecular responses during the secondary brain injury phase. Also, since brain glucose metabolism is an orchestrated interplay, measuring the expressions of the above-mentioned genes simultaneously will help to identify which steps of glucose utilization might be affected by mTBI.

However, studying the chronic effects of mTBI is challenged by the lack of a suitable rodent model with prolonged symptoms similar to those in patients with mTBI. In most mTBI rodent models, the majority of behavioral abnormalities recover spontaneously within 3–4 weeks or have not been assessed for longer than that period (28–32). To establish an animal model of mTBI with neurobehavioral abnormalities that can last for months, we used repetitive mild closed head impacts to induce the brain injury in mice. These mice exhibited several neurobehavior impairments for at least 6 months post brain injury. This mouse model of mTBI allowed us to investigate damaged brain at molecular level concomitant neurobehavioral abnormalities during the chronic phase of mTBI.

We chose 10, 19, and 28 weeks post mTBI as time points (Figure 1B) for neurobehavioral measurements and assessed gene expression in cortex and hippocampus for both ipsilateral and contralateral sides, hypothalamus, brainstem, and cerebellum. These brain areas were either directly or indirectly injured by repetitive impacts and represented brain areas that were at different distances adjacent to a directly injured ipsilateral cortex. They also play different roles in neurobehavioral performance.

The aforementioned experimental design allowed us to examine glucose metabolism—related gene expression in different brain regions, concomitant with neurobehavioral impairments, during the chronic phase of mTBI.

## Materials and Methods

### Animals and Repetitive Mild Closed Head Brain Injury

All protocols (protocol number #01701) were approved by the Washington DC VA Medical Center's Animal Care and Use, Research Safety and Research and Development Committees, and followed National Institutes of Health standards.

**Figure 2:**
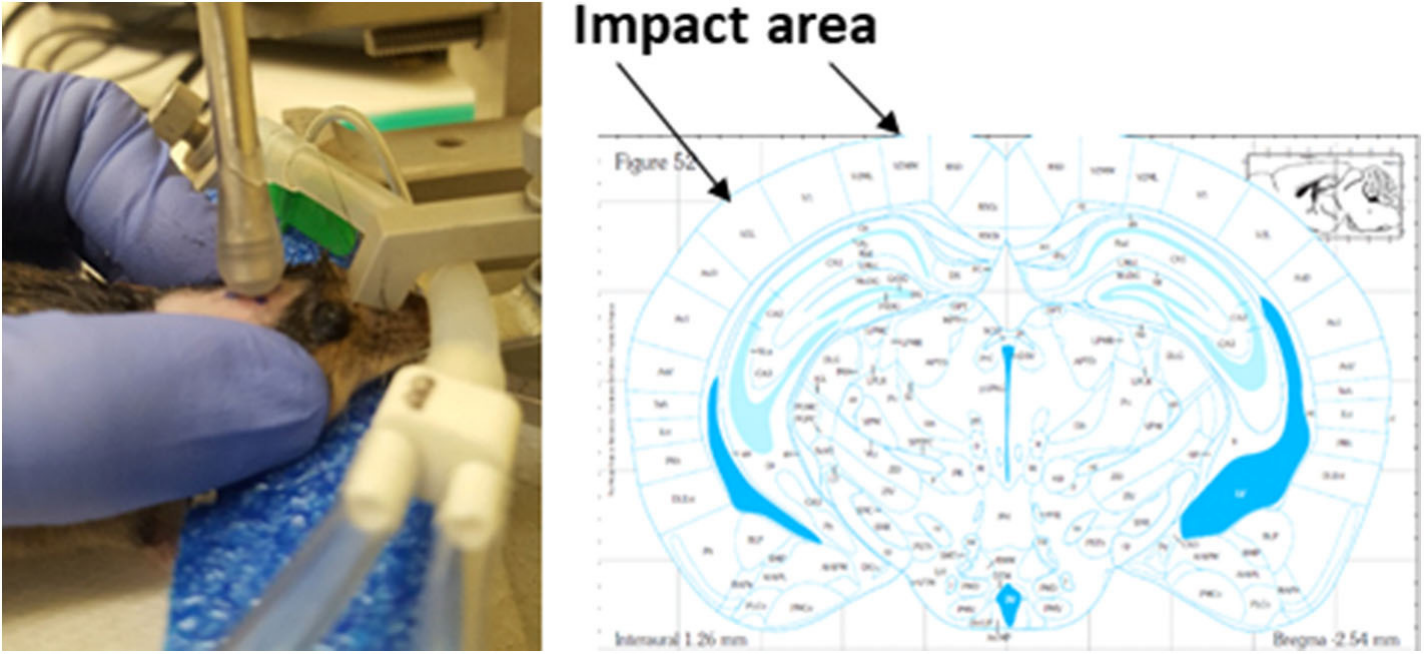
Demonstration of brain impact setting and brain area of injured.

Healthy, intact male C57BL/6J mice (9 weeks old from Jackson Laboratories, Bar Harbor, ME) were group housed at room temperature (22 ± 1°C) with a 12-h light/dark cycle and had free access to tap water and rodent chow (Teklad rodent chow diet 8604, Envigo RMS Division, Indianapolis, IN 46250 United States). During a housing adaptation week, the heads of mice were shaved by hair removal lotion and the impact area was marked using a sharpie permanent marker (www.sharpie.com). By the end of the adaptation period, mice were randomly divided into sham and mTBI groups (*n* = 45/group) and were anesthetized through a nose mask using Isoflurane (induction at 4% and maintenance at 1–2%) delivered by SomnoSuite™ Small Animal Anesthesia System (Kent Scientific. Torrington CT 06790 USA). Anesthesia depth was monitored by assessing pedal withdrawal reflexes. Isoflurane anesthetized mice were then placed on a stereotaxic frame without ear bars, but with a palate bar and nose clamp. The custom-made polyethylene foam (1.9” high) was also placed under the mouse head for additional support. Immediately after each mouse's pedal withdrawal reflexes reoccurred following light anesthesia, the head injury was delivered using a controlled cortical impact device (Leica MyNeuroLab Impact One™ Stereotaxic Impactor, Leica Biosystems Richmond Inc. Richmond IL 60071 USA) with custom made rubber tip (5.0 mm diameter, round shape) mounted directly on a stereotaxic instrument. This device uses electromagnetic force to produce an impact velocity with speed, depth, and dwell time all being individually manipulated to produce accurate and precise severity of injuries. The impact area included 2~4 mm left of midline and−1.0~-3.0 mm from bregma on the cortex, located directly above the left hippocampus. Impact velocity and dwell time were 5.25 m/s and 0.1 s, respectively. The impact depth was 2.0 mm. Identical impacts were repeated every two days for a total of five times over a two-week period. Sham operated mice underwent the same procedure, but the impact was delivered into the air, not to the brain (Figure 2). Of note, when the impacts were applied, mice had already regained pedal withdrawal reflexes. This setting was intended to mimic the situation found in humans, as no anesthesia is ordinarily available when humans experience brain injury. All mice in the mTBI group survived repeated impacts. Among total of 225 impacts (5 impacts for each mouse for total of 45 mice in mTBI group), 15% of impacts did not produce immediate walking/moving abnormalities in mice, and 81% of impacts made mice had transient loss (<1 min) of pedal withdrawal reflexes.

### Experiment Schedule and Brain Tissue Collection

After completion of the brain injury (week 0), mice were divided into three cohorts and each cohort (*n* = 15/group for total of 30 mice) was tested at one of three time periods described below, followed by brain tissue collection for post-mortem measurements. Morris water maze (MWM), elevated plus maze (EPM), and neurological severity score test (NSS) were used for testing neurobehavioral deficits. MWM, EPM, and NSS, were conducted at 8, 9, and 10 weeks for the 1st time period, 17, 18, and 19 weeks for the 2nd time period, and 26, 27, and 28 weeks for the 3rd time period, respectively. Thus, mice were not repeatedly tested for the same behavioral assessment. At each time point and two days after the NSS test, among the 15 mice in each group that were used for the neurobehavioral tests, 10 mice were decapitated for brain tissue collection and 5 mice were transcardially perfused with 0.9% saline solution, followed by 4% PFA solution. After the post-fixation step, the brains were cryoprotected with a 30% sucrose solution. The experimental schedule is schematically illustrated in Figure 1B.

### Behavioral Testing

All behavioral testing was conducted during the light cycle phase and in enclosed behavior rooms adjacent to the room where mice were housed. The mice were placed in testing rooms for 30 min for acclimation prior to the onset of behavioral testing. MazeScan Basic and Topscan LITE software (Clever Sys. Incorporated. Reston, VA USA 20190) were used to record and analyze MWM and EPM data.

#### MWM

The circular water tank pool (48” diameter, San Diego Instruments) was filled with 20°C tap water with opaque nontoxic white tempera paint added. Custom-made visual cues were hung on the walls surrounding the pool. The clear plastic platform (4” diameter, round shape) was placed 0.5 cm below the water surface in one of the four quadrants of the pool (referred to as “correct quadrant”) and remained constant during the spatial acquisition testing paradigm. All trails were recorded by video camera positioned above the maze. Mouse was facing the wall of tank away from the pool and was released by experimenter from one of the three different quadrants that do not contain the platform. Mouse was allowed to find the hidden platform within 90 s period. The time used for each mouse to reach the platform was recorded as the escape latency. Each mouse was tested on three trails per day for 5 consecutive days (Training trail) as spatial learning test. The average of three recorded escape latency was used for analysis. The probe trail was conducted 72 h after the last training trail day to evaluate the memory retention. On probe trail day, the platform was removed from the pool and the mice were released in the water tank from the quadrant opposite from the correct quadrant and allowed to swim for 30 s. The travel time (seconds) for each mouse in current quadrant was recorded and used as indicator of memory retention.

#### EPM

The EPM apparatus (San Diego Instruments) is made of non-porous blue plastic. It consists of two sets of cross-shaped arms that elevated 21.25” above the ground. The width and length of arms are 2” and 26” respectively. One set of arms is enclosed with walls (closed arms), and the other is exposed (open arms). Because mice have a natural preference for dark enclosed spaces, changes in neurobehavior activities were detected by quantifying the time spent in both arms for each mouse to assess the presence of anxiety-related or risk-taking behaviors. Each mouse was released at the center junction of the maze facing an open arm that was opposite to where the experimenter was standing and were given 5 min to explore. The time spent in each arm and number of entries into each arm were recorded by video camera positioned above the maze and analyzed. Ratio of duration spent on open/closed arms was calculated as an indicator of fearless condition for mice. The total motor activity was calculated as the sum of the recorded number of times each mouse entered the open, closed and center area in the EPM apparatus during the 5 min testing period.

#### NSS

Neurological severity score (NSS) is an assessment of posttraumatic neurological impairment, commonly used during the acute phase of TBI (33, 34). The assessment is composed of 10 different tasks, including tasks on motor function, alertness, and certain physiological behaviors. Each mouse was given 1 point for failure of the task and 0 point for succeeding. Therefore, a score of 10 represents mouse failure in all tasks, an indicator of severe neurological dysfunction; and a score of 0 indicates a healthy mouse or that a mouse has recovered from injury for these particular neurobehavioral performances (34). In the present study, the NSS was assessed by an investigator who was blinded to the experimental animal groups. Data are presented as mean values ± SE (*n* = 14–15/group).

### Histology

The brains were cryopreserved with a 30% sucrose solution and were cut in 40 μm thickness coronal sections from 1.18 mm to −4.48 mm from Bregma at 320 μm interval. The sections were processed for detection of neurodegeneration with FD NeuroSilver™ Kit II (FD Neurotechnologies, Ellicott City, MD). All samples were processed by FD NeuroTechnologies INC according to the manufacturer's instructions.

The sections were mounted on microscope slides and cover-slips with Permount (Fisher Scientific, Fair Lawn, NJ). Investigators blinded to the injury conditions took images of the sections using an inverted microscope ZEISS MCU 2008 and AxioVision 4 software at uniform criteria for sensitivity and exposure time. To ensure objective quantification, the same threshold value was applied to all brain sections. The edge of the corpus callosum (CC) was traced on the image of the same section for both sham and injured mice to obtain the area. The mean gray value in the area of the CC was used as an indicator of stain intensity. All quantification was performed by an investigator blinded to the groups using Image J (NIH, Version 2).

### Gene Expression Measurements

Immediately after decapitation, mouse brains were dissected on ice for collection of the following brain tissues: parietal frontal cortex and hippocampus both ipsilateral and contralateral to the sides of injury, brainstem, hypothalamus, and cerebellum. All brain tissues were immediately placed in RNAlater® Solution (Ambion, Thermo Fisher Scientific) and stored at −20°C. Total RNA was extracted from brain tissues using Trizol reagent.

Verifications were performed to ensure accuracy of the real-time PCR TaqMan^TM^ method, as we published previously (35). The detailed real-time PCR TaqMan^TM^ conditions were the same as per the protocol provided by manufacture. The probes for each target gene and cyclophilin were labeled with FAM and VIC, respectively. The expressions of 10 different genes (Table 1) in the above-mentioned brain tissue were measured by the multiplex q-PCR method. A comparative Ct (ddCt) method was used to determine gene expression levels.

**Table 1:** Brain glucose/lactate utilization related genes.

**Gene name**	**TaqMan^**TM**^ gene expression ID**	**Function**
HK1	Mm00439344_m1	Hexokinase 1
PFK	Mm01309576_m1	Phosphofructokinase
PK	Mm00834102_gH	Pyruvate kinase
PDH	Mm00499323_m1	Pyruvate dehydrogenase
Slc2a1	Mm00441480_m1	Glut-1: capillary glucose transporter
Slc2a3	Mm00441483_m1	Glut-3: neuron glucose transporter
Slc16a1	Mm01306379_m1	MCT-1: astrocyte lactate transport
Slc16a7	Mm00441442_m1	MCT-2: neuron lactate transporter
GPR81	Mm00558586_s1	lactate receptor
Hk2	Mm00443385_m1	Hexokinase isoform 2

Results are expressed as the % change over their corresponding control samples, which were the same brain regions collected at 9-week post injury from the sham group. If both ipsilateral and contralateral brain regions were collected, the contralateral sites collected at 9 weeks post injury in sham group were used as control samples.

### Statistical Analysis

Data are presented as mean values ± SEM. *P* < 0.05 was considerate statistically significant.

SAS 9.2 was used for two-way repeated measures ANOVA to analyze MWM escape latency with day as the repeated measure and treatment (sham vs. mTBI) as another independent variable. For the MWM probe trail, the travel time in the correct quadrant was analyzed by *T*-test using an Excel data analysis tool.

The Microsoft excel data analysis tool was used for Two-way ANOVA with treatment (sham vs. mTBI) and time post mTBI (10th, 19th, 28th weeks post mTBI) as the two independent variables for the following analyses: (1) the proportion of time spent on open/closed arms and motor activity for EMP, (2) NSS data, (3) Silver stain data, and (4) gene expression data.

The gene expression data were analyzed for each gene at each brain tissue separately and there was no comparison among different genes or different brain tissues. As we were interested in two main effects (time and mTBI) over the entire study period rather than for each individual time point, we did not do *post hoc* analysis to compare differences at each individual time point. Only when the interaction (treatment X time) became significant the mTBI group was compared with the sham group at that particular time point for that specific gene. The detailed statistical parameters such as F value and “n” for each analysis are provided in Supplementary Files 1–5.

## Results

### Behavior Test Results

#### MWM

**Figure 3:**
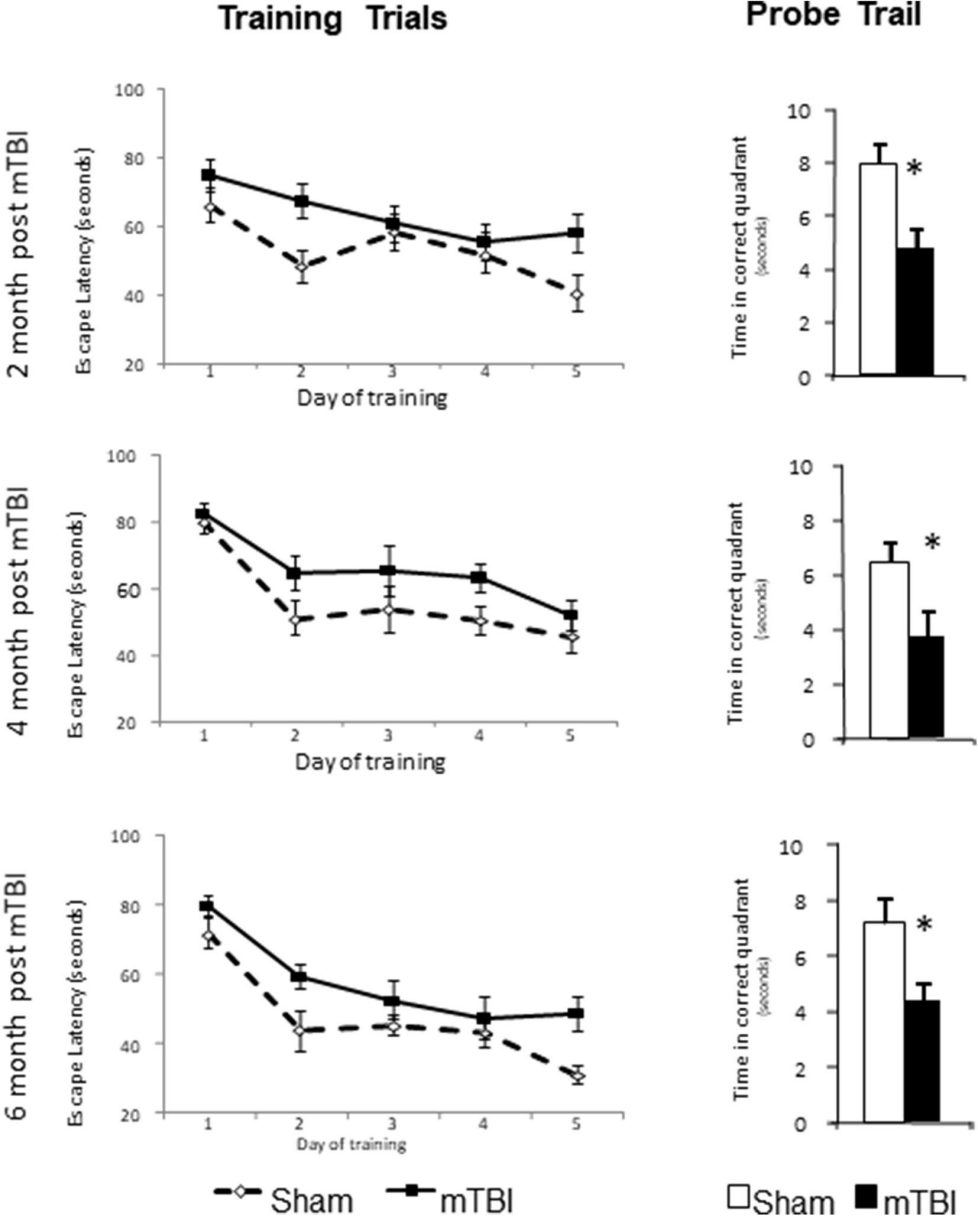
mTBI lead to learning and memory deficits in C57bl/6J mice determined by Morris water maze at 2-, 4-, and 6-month after the injury. Mice had training trail from day 1–5 and probe trail at day 8. Data are mean values ± SE, *n* = 15 per group. For all leaning trails, Time effect: *P* < 0.01, TBI effect: *P* < 0.05, Time X TBI Interaction: >0.05. For all probe trails: *P < 0.05 compared to sham injured mice.

The training trails and probe trails are commonly used for testing spatial learning and memory retention in mice. Repeated measurements were used for comparison of escape latencies among five days training trails (time effect) and between sham and mTBI groups (injury effect) and the interaction of these two effects. A significant time effect (*P* < 0.01) in all training trails was observed, which indicates that the experimental setting was valid and that mice were learning during the training trails. However, mice in the mTBI group experienced a longer escape latency (*P* < 0.05) to reach the hidden platform in 5 days training trails and shorter time spent in the correct quadrant in probe trail (*P* < 0.05) when compared with the Sham injured group at all three testing periods. There was no significant interaction between the time and treatment effects. For the probe trails, mTBI groups spent significantly less time in the correct quadrant at all three time points tested (*P* < 0.05). The time spent in the correct quadrant for sham groups were 7.92 ± 0.78, 6.41 ± 0.79, and 7.18 ± 0.92 s at 2-, 4-, and 6-months post mTBI, respectively. We notice that the time spent in the correct quadrant was <¼ of 30 s testing period for sham groups at 4- and 6- months post mTBI. The poor performance of the sham group might have been due to the aging process or to other unknown confounding factors. Regardless, the mTBI group performed much worse than the sham group at all the same time points. These results indicate impaired learning and memory for mTBI mice at 2-, 4-, and 6- months post mTBI (Figure 3).

#### EPM

**Figure 4:**
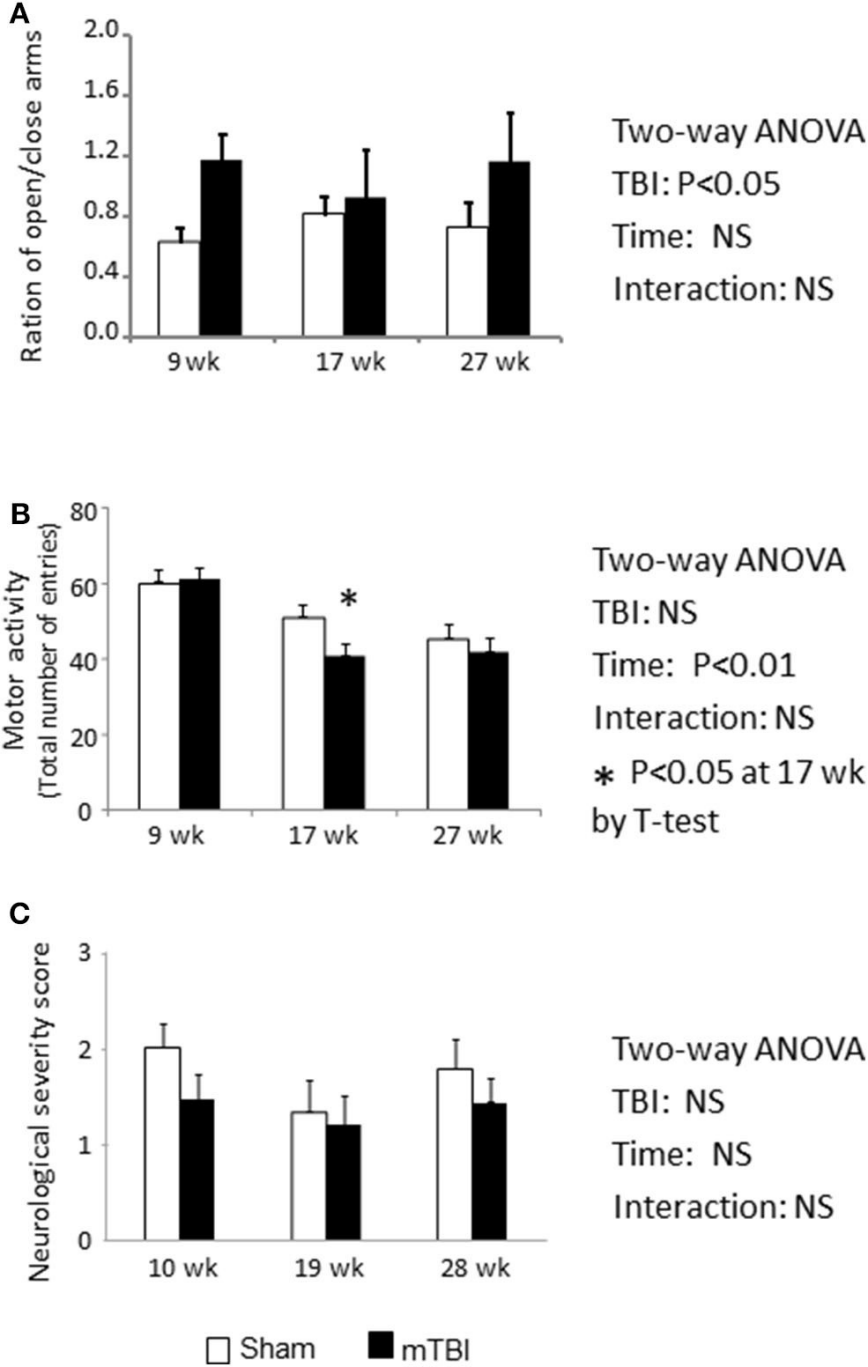
EPM test indicate Ration of duration spent on open/closed arms was increased in mTBI mice **(A)**. Total motor activity was decreased by the aging (*P* < 0.01) and mTBI group has accelerated the decreases at 18-week post mTBI (*P* < 0.05) **(B)**. NSS was not altered by either mTBI or aging **(C)**. Data are mean values ± SE (*n* = 15/group) * indicates *P* < 0.05 when compared with the sham group at the same time point.

Normal mice naturally prefer to stay in closed arms compared to open arms. During a 5 min EPM testing period, mice in the mTBI group exhibited an overall higher proportion of time on open vs. closed arms (*P* < 0.05 by Two-way ANOVA); the *post hoc* group comparison indicated that this effect mainly resulted from measurements conducted during the first time period (9th weeks post injury) (Figure 4A). This higher proportion of time spent in the open vs. closed arms might indicate that the TBI mice exhibited increased risk-taking behavior.

During the 6 month study period, the number of total entries was significantly decreased in both the sham and mTBI groups (Figure 4B. Time effect: *P* < 0.01), possibly resulting from reduction in activity due to aging. However, mice in the mTBI group exhibited an accelerated reduction in total entries compared with the sham group, as indicated by a lower number of total entries than in sham mice at 18 weeks post injury (*P* < 0.05) (Figure 4B).

#### NSS

The neurological severity score is commonly used to assess neurological impairments during the acute phase of TBI (33). When mice were examined at 10, 19, 28 weeks post mTBI, NSSs were not statistically different between the sham and mTBI groups, and both group's scores were within the normal range (Figure 4C).

### Histology

#### Sliver Stain

**Figure 5:**
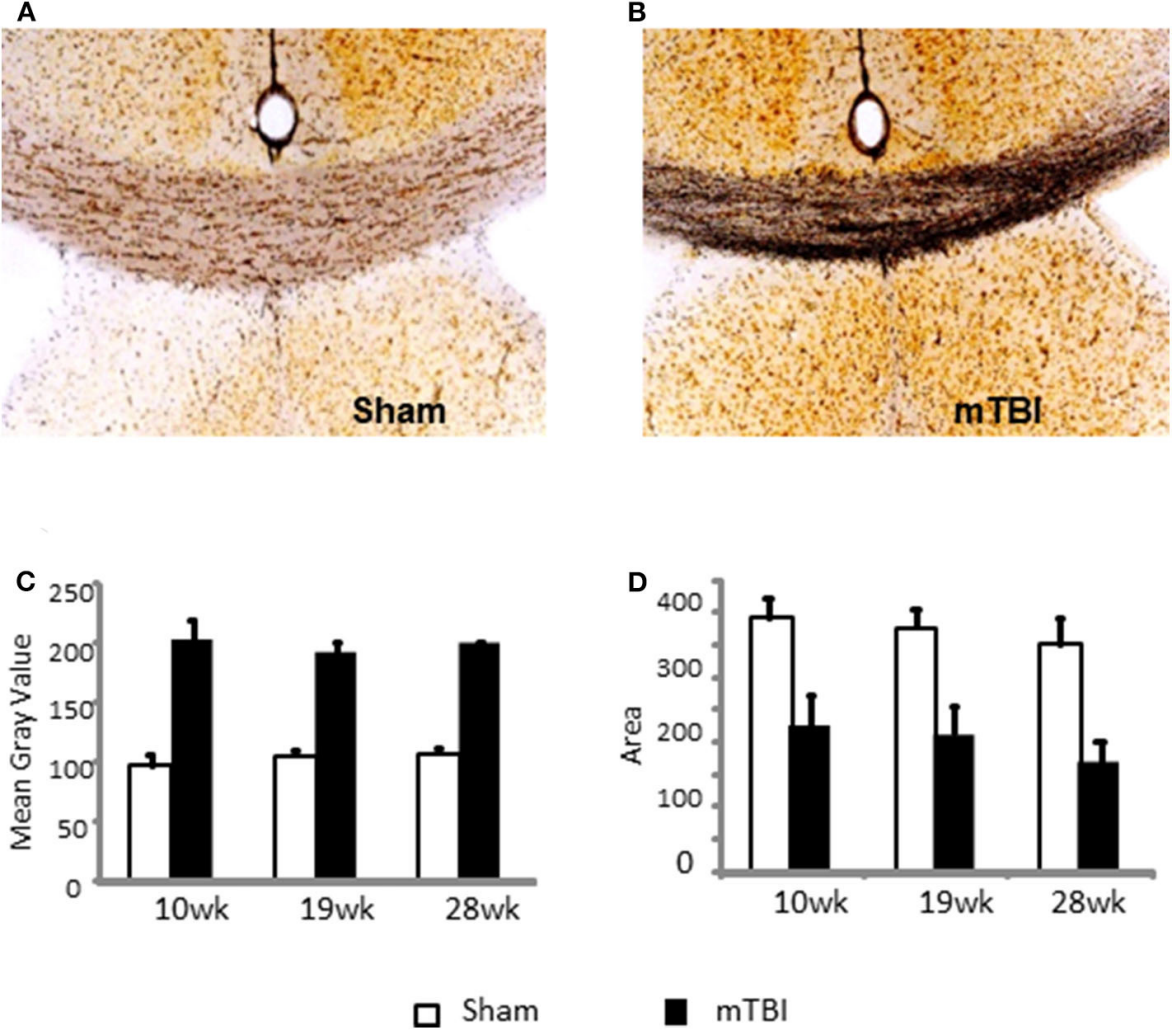
Quantification of neurodegeneration by NeuroSilver™ Kit in the corpus callosum (CC) in sham **(A)** and mTBI **(B)** mice mTBI significantly increased degenerating neurons in CC **(C)** and decreased area of CC **(D)**. Data are mean values ± SE (*n* = 5 for each group at each time point). For both mean gray area and area of CC, *P* < 0.01 for effect of mTBI. The effects of time and time X injury interaction were not significant.

Representative images are demonstrated in Figures 5A,B for sham and mTBI mice, respectively. Quantification of neurodegeneration by silver staining in the corpus callosum (CC) demonstrated long-lasting neuronal damage in mTBI mice. The mean gray value of staining in CC was significantly increased (*P* < 0.01, Figure 5C) and the area of CC was significantly decreased (*P* < 0.01 Figure 5D) in the mTBI groups at all three time points. The effects of time and time X mTBI interaction were not significant. These data, in association with long-term declines in neurobehavior function, revealed chronic impairments in our mouse model of mTBI.

### Gene Expression

Gene expression data were analyzed by two-way ANOVA for each individual brain tissue tested. The two independent variables were TBI effect (sham vs. mTBI treatment) and time effect (10, 19, and 28 weeks post mTBI). The “TBI effect” represented overall effect of mTBI vs. Sham, and “Time effect” represented whether gene expression changed with time during the study period. As our measurements spanned from 2 months to over 6 months post completion of mTBI, the time effect can also be considered an “aging” effect.

Additionally, of the 10 mRNAs measured, eight mRNAs encode important molecules in brain glucose transport and metabolism: HK1, PFK, PK, PDH, Glut-1, Glut-3, MCT-1, and MCT-2 (Figure 1). The other two, HK2 and GPR81, encode molecules that can be used as indicators for abnormal brain glucose utilization (24–26).

Because of the large amount of data, detailed expression results for each gene are summarized in Table 2 for the hippocampus, Table 3 for the cortex, Table 4 for the hypothalamus, Table 5 for the brainstem, and Table 6 for the cerebellum. Only *P* values that are < 0.05 (statistically significant) are listed on the tables. The detailed data and statistical analysis parameters are presented as Supplementary Files 1–5.

**Table 2:** Genes expression in hippocampus of sham and mTBI mice.

**Gene**	**Tissue**	**Treat**	**Time post mTBI**			**P value**		
			**Week 10**	**Week 19**	**Week 28**	**TBI effect**	**Time effect**	**TBI X time interaction**
HK1	Contralateral cortex	Sham	100.0 ± 5.1	85.9 ± 2.7	92.2 ± 2.9		<0.05	
		mTBI	99.5 ± 2.8	92.9 ± 5.1	94.8 ± 3.2			
	Ipsilateral cortex	Sham	95.8 ± 3.1	87.7 ± 2.3	96.2 ± 3.3		<0.01	<0.05
		mTBI	88.2 ± 8.5^*^	84.0 ± 5.0	109.7 ± 7.0			
PFK	Contralateral cortex	Sham	100.0 ± 4.0	86.4 ± 3.1	93.4 ± 2.3		<0.01	
		mTBI	97.7 ± 5.5	85.6 ± 3.3	96.6 ± 3.2			
	Ipsilateral cortex	Sham	89.5 ± 3.4	81.8 ± 2.2	95.2 ± 1.3		<0.01	
		mTBI	87.0 ± 2.7	85.0 ± 3.8	106.9 ± 3.6			
PK	Contralateral cortex	Sham	100.0 ± 8.5	74.9 ± 4.5	70.8 ± 7.0	<0.01	<0.01	
		mTBI	84.0 ± 9.7	56.8 ± 6.6	48.7 ± 6.2			
	Ipsilateral cortex	Sham	52.6 ± 2.6	42.7 ± 3.4	28.5 ± 2.5	<0.01	<0.01	<0.05
		mTBI	55.6 ± 4.5	31.0 ± 4.3^*^	16.0 ± 2.6^**^			
PDH	Contralateral cortex	Sham	100.0 ± 8.6	52.6 ± 6.4	40.2 ± 4.7	<0.05	<0.01	
		mTBI	96.2 ± 6.4	38.0 ± 4.3	29.5 ± 3.5			
	Ipsilateral Cortex	Sham	48.3 ± 4.0	44.0 ± 7.5	28.0 ± 4.5	<0.05	<0.01	
		mTBI	40.6 ± 3.7	37.8 ± 5.5	16.3 ± 3.6			
Glut-1	Contralateral cortex	Sham	100.0 ± 4.1	61.1 ± 3.3	71.1 ± 4.9		<0.01	
		mTBI	105.2 ± 6.6	64.3 ± 4.5	75.7 ± 3.7			
	Ipsilateral cortex	Sham	89.4 ± 5.9	63.7 ± 3.4	71.2 ± 5.1		<0.01	
		mTBI	86.4 ± 4.0	58.6 ± 3.5	64.6 ± 2.9			
Glut-3	Contralateral cortex	Sham	100.0 ± 4.3	76.9 ± 2.6	84.1 ± 2.5		<0.01	
		mTBI	97.2 ± 2.9	81.2 ± 4.1	89.4 ± 4.3			
	Ipsilateral cortex	Sham	80.6 ± 6.3	78.8 ± 3.4	93.7 ± 2.2		<0.01	
		mTBI	73.0 ± 2.3	78.2 ± 3.0	100.3 ± 5.0			
MCT-1	Contralateral cortex	Sham	100.0 ± 3.6	65.9 ± 3.3	68.3 ± 4.4		<0.01	
		mTBI	99.6 ± 5.6	71.8 ± 7.9	71.5 ± 4.4			
	Ipsilateral cortex	Sham	78.7 ± 4.5	61.5 ± 4.1	75.1 ± 3.0		<0.01	
		mTBI	75.2 ± 2.7	68.8 ± 4.5	72.0 ± 7.9			
MCT-2	Contralateral cortex	Sham	100.0 ± 4.6	67.6 ± 3.1	70.4 ± 5.0		<0.01	
		mTBI	93.5 ± 3.4	68.7 ± 3.4	68.8 ± 3.9			
	Ipsilateral cortex	Sham	73.3 ± 4.5	65.4 ± 2.8	78.3 ± 3.1			
		mTBI	73.0 ± 3.3	66.4 ± 3.9	64.8 ± 3.7			
HK2	Contralateral cortex	Sham	100.0 ± 5.4	85.2 ± 5.9	82.9 ± 7.8	<0.01		
		mTBI	117.9 ± 11.9	109.4 ± 7.6	102.7 ± 5.9			
	Ipsilateral cortex	Sham	94.3 ± 5.3	82.6 ± 7.3	97.3 ± 6.6	<0.01	<0.05	
		mTBI	104.7 ± 11.6	98.2 ± 10.1	131.9 ± 10.4^*^			
GPR81	Contralateral cortex	Sham	100.0 ± 14.8	90.6 ± 10.8	74.0 ± 9.5	<0.01		
		mTBI	161.2 ± 40.5	139.7 ± 22.8	134.5 ± 21.4			
	Ipsilateral cortex	Sham	86.8 ± 13.0	88.1 ± 19.7	77.4 ± 8.5	<0.05		
		mTBI	107.4 ± 17.8	141.6 ± 23.3	92.2 ± 6.6			

*Data are presented as % change of expression level relative to the contralateral side of the hippocampus in sham group measured at week 10 post mTBI*.

*Data are mean ± SE (n = 10/group)*.

*P Value: Obtained from Two-Way ANOVA analysis. If P > 0.05, the value is not listed on the table*.

* P < 0.05 and

** *P < 0.01 when compared with the Sham group at the same injury sides and same time point by T-test*.

**Table 3:** Genes expression in Cortex of sham and mTBI mice.

**Gene**	**Tissue**	**Treat**	**Time post mTBI**			***P*** **value**		
			**Week 10**	**Week 19**	**Week 28**	**TBI effect**	**Time effect**	**TBI X time interaction**
HK1	Contralateral cortex	Sham	100.0 ± 8.7	87.8 ± 8.2	55.3 ± 3.0		<0.01	
		mTBI	92.9 ± 6.6	67.5 ± 4.3	57.5 ± 3.1			
	Ipsilateral cortex	Sham	103.2 ± 8.6	108.1 ± 7.0	97.7 ± 4.0			
		mTBI	101.4 ± 8.5	102.2 ± 6.7	87.9 ± 7.6			
PFK	Contralateral cortex	Sham	100.0 ± 3.0	109.9 ± 6.3	95.0 ± 3.6			<0.05
		mTBI	95.7 ± 5.5	90.1 ± 2.2^**^	97.2 ± 2.0			
	Ipsilateral cortex	Sham	98.3 ± 5.0	95.8 ± 4.1	102.9 ± 2.7			
		mTBI	107.2 ± 3.9	89.5 ± 10.7	99.2 ± 5.3			
PK	Contralateral cortex	Sham	100.0 ± 5.3	103.5 ± 4.6	111.6 ± 3.9		<0.01	
		mTBI	97.0 ± 5.9	97.1 ± 3.5	112.5 ± 3.7			
	Ipsilateral cortex	Sham	90.3 ± 5.0	103.1 ± 2.9	104.0 ± 5.4			
		mTBI	96.5 ± 4.4	104.0 ± 5.5	103.1 ± 5.1			
PDH	Contralateral cortex	Sham	100.0 ± 9.0	88.2 ± 7.4	71.5 ± 4.9		<0.01	
		mTBI	86.5 ± 8.8	67.9 ± 3.7	72.3 ± 4.7			
	Ipsilateral cortex	Sham	98.4 ± 9.3	93.1 ± 6.5	98.8 ± 4.5			
		mTBI	95.9 ± 8.7	96.2 ± 5.3	88.4 ± 6.7			
Glut-1	Contralateral cortex	Sham	100.0 ± 5.3	86.3 ± 5.8	100.9 ± 5.2		<0.05	
		mTBI	90.0 ± 4.0	83.2 ± 2.5	96.5 ± 4.5			
	Ipsilateral cortex	Sham	89.0 ± 3.2	91.5 ± 6.8	89.5 ± 3.8			
		mTBI	90.6 ± 3.8	93.0 ± 6.5	87.2 ± 5.3			
Glut-3	Contralateral cortex	Sham	100.0 ± 6.3	89.2 ± 4.4	68.0 ± 3.2		<0.01	
		mTBI	88.3 ± 5.2	78.8 ± 3.3	71.3 ± 3.2			
	Ipsilateral cortex	Sham	90.8 ± 3.7	92.0 ± 5.6	89.1 ± 3.7		<0.05	
		mTBI	92.2 ± 5.7	100.3 ± 7.3	76.4 ± 5.0			
MCT-1	Contralateral cortex	Sham	100.0 ± 14.7	78.9 ± 8.0	101.9 ± 10.1			
		mTBI	78.0 ± 5.8	93.5 ± 4.5	81.8 ± 4.3			
	Ipsilateral cortex	Sham	82.0 ± 5.1	86.0 ± 8.8	91.8 ± 6.3			<0.05
		mTBI	86.4 ± 5.8	97.2 ± 12.0	66.2 ± 5.5^**^			
MCT-2	Contralateral cortex	Sham	100.0 ± 7.2	74.2 ± 4.0	62.9 ± 8.0		<0.01	
		mTBI	101.6 ± 5.4	67.9 ± 4.4	54.5 ± 8.1			
	Ipsilateral cortex	Sham	94.4 ± 9.9	73.9 ± 10.9	69.8 ± 4.4		<0.05	
		mTBI	85.1 ± 6.9	73.2 ± 9.2	60.6 ± 6.0			
HK2	Contralateral cortex	Sham	100.0 ± 6.5	90.5 ± 10.9	59.8 ± 3.6		<0.01	
		mTBI	83.4 ± 11.5	83.4 ± 10.8	60.4 ± 3.2			
	Ipsilateral cortex	Sham	77.8 ± 7.8	95.2 ± 10.3	98.3 ± 5.0		<0.05	<0.05
		mTBI	90.8 ± 8.2	112.9 ± 12.8	69.9 ± 6.5^**^			
GPR81	Contralateral cortex	Sham	100.0 ± 19.3	90.5 ± 15.8	60.2 ± 7.1		<0.01	
		mTBI	122.1 ± 23.3	80.3 ± 11.2	54.6 ± 8.3			
	Ipsilateral cortex	Sham	94.5 ± 15.2	110.5 ± 16.4	69.4 ± 12.8		<0.01	
		mTBI	84.3 ± 17.2	121.7 ± 18.3	54.3 ± 9.6			

*Data are presented as % change of expression level relative to the contralateral side of the cortex in the sham group measured at week 10 post mTBI*.

*Data are mean ± SE (n = 9–10/group)*.

*P Value: Obtained from Two-Way ANOVA analysis. If P>0.05, the value is not listed on the table*.

** *P < 0.01 when compared with the Sham group at the same injury sides and same time point by T-test*.

**Table 4:** Genes expression in hypothalamus of sham and mTBI mice.

**Gene**	**Treat**	**Time post mTBI**			***P*** **value**		
		**Week 10**	**Week 19**	**Week 28**	**TBI effect**	**Time effect**	**Interaction**
HK1	Sham	100.0 ± 3.0	114.2 ± 5.8	91.2 ± 7.7		<0.01	
	mTBI	102.9 ± 4.0	110.8 ± 4.0	100.6 ± 3.4			
PFK	Sham	100.0 ± 3.5	112.4 ± 3.8	84.8 ± 6.9		<0.01	
	mTBI	98.2 ± 3.2	103.6 ± 3.6	94.3 ± 2.2			
PK	Sham	100.0 ± 8.1	89.6 ± 7.5	73.1 ± 6.2		<0.01	
	mTBI	101.9 ± 4.9	98.2 ± 5.3	83.5 ± 6.1			
PDH	Sham	100.0 ± 7.3	95.2 ± 6.1	91.7 ± 4.8			
	mTBI	94.9 ± 6.0	87.4 ± 6.3	78.3 ± 3.4			
Glut-1	Sham	100.0 ± 3.8	119.9 ± 7.4	100.7 ± 3.4			
	mTBI	104.1 ± 4.8	108.1 ± 6.9	105.7 ± 6.9			
Glut-3	Sham	100.0 ± 3.1	114.2 ± 4.0	100.5 ± 3.8		<0.05	
	mTBI	101.0 ± 4.4	106.8 ± 4.2	104.3 ± 2.5			
MCT-1	Sham	100.0 ± 5.3	120.2 ± 4.0	98.1 ± 8.2			
	mTBI	112.6 ± 4.8	113.4 ± 8.8	108.2 ± 3.8			
MCT-2	Sham	100.0 ± 6.2	114.9 ± 6.9	98.5 ± 6.8		<0.01	
	mTBI	104.8 ± 4.2	125.8 ± 9.9	100.9 ± 3.9			
HK2	Sham	100.0 ± 9.1	103.9 ± 8.4	76.7 ± 3.5	<0.01	<0.05	
	mTBI	120.4 ± 9.4	127.0 ± 6.5	115.9 ± 6.6			
GPR 81	Sham	100.0 ± 22.6	84.7 ± 10.4	104.4 ± 14.7			
	mTBI	101.7 ± 10.8	88.3 ± 14.9	86.4 ± 12.7			

*Data are presented as % change of expression level in the hypothalamus of the sham group measured at week 10 post mTBI*.

*Data are mean ± SE (n = 9-10/group)*.

*P Value: Obtained from Two-Way ANOVA analysis. If P > 0.05, the value is not listed on the table*.

**Table 5:** Genes expression in brainstem of sham and mTBI mice.

**Gene**	**Treat**	**Time post mTBI**			**P value**		
		**Week 10**	**Week 19**	**Week 28**	**TBI effect**	**Time effect**	**Interaction**
HK1	Sham	100.0 ± 10.2	117.3 ± 11.2	95.7 ± 5.9			
	mTBI	114.1 ± 13.2	107.7 ± 5.1	97.7 ± 4.5			
PFK	Sham	100.0 ± 6.6	109.8 ± 6.4	109.3 ± 4.2			
	mTBI	108.9 ± 6.42	113.71 ± 4.5	114.9 ± 5.1			
PK	Sham	100.0 ± 8.5	93.3 ± 5.0	86.1 ± 4.1		<0.01	
	mTBI	105.8 ± 5.8	100.7 ± 5.0	86.6 ± 4.3			
PDH	Sham	100.0 ± 6.2	94.6 ± 3.6	86.1 ± 6.1		<0.05	
	mTBI	115.0 ± 9.2	94.5 ± 5.7	99.8 ± 5.4			
Glut-1	Sham	100.0 ± 8.0	107.2 ± 6.1	73.7 ± 3.5		<0.01	
	mTBI	100.3 ± 8.5	99.2 ± 4.0	74.9 ± 3.6			
Glut-3	Sham	100.0 ± 9.6	107.1 ± 7.0	81.2 ± 4.4		<0.01	
	mTBI	105.9 ± 6.6	94.5 ± 2.7	86.7 ± 2.5			
MCT-1	Sham	100.0 ± 8.2	115.5 ± 10.1	86.7 ± 5.6		<0.01	
	mTBI	108.3 ± 6.7	116.9 ± 6.9	97.2 ± 5.8			
MCT-2	Sham	100.0 ± 15.8	102.4 ± 17.8	64.8 ± 3.5		<0.01	
	mTBI	118.4 ± 12.1	87.6 ± 6.9	76.5 ± 6.5			
HK2	Sham	100.0 ± 9.9	97.8 ± 15.9	86.1 ± 4.0	<0.05	<0.05	
	mTBI	130.7 ± 14.4	134.6 ± 13.8	92.1 ± 5.8			
GPR 81	Sham	100.0 ± 19.5	94.7 ± 21.2	63.8 ± 13.9	<0.01	<0.01	
	mTBI	225.2 ± 35.2	154.8 ± 23.1	97.87 ± 21.9			

*Data are presented as % change of expression level in the brainstem of the sham group measured at week 10 post mTBI*.

*Data are mean ± SE (n = 9-10/group)*.

*P Value: Obtained from Two-Way ANOVA analysis. If P > 0.05, the value is not listed on the table*.

**Table 6:** Genes expression in cerebellum of sham and mTBI mice.

**Gene**	**Treat**	**Time post mTBI**			***P*** **value**		
		**Week 10**	**Week 19**	**Week 28**	**TBI effect**	**Time effect**	**Interaction**
HK1	Sham	100.0 ± 8.4	107.8 ± 10.0	93.5 ± 9.6			
	mTBI	111.2 ± 10.3	86.2 ± 5.9	99.9 ± 9.5			
PFK	Sham	100.0 ± 4.1	107.4 ± 9.3	93.0 ± 5.0			
	mTBI	101.7 ± 5.6	93.7 ± 3.7	108.7 ± 8.3			
PK	Sham	100.0 ± 13.1	154.4 ± 15.8	259.0 ± 19.6	<0.05	<0.01	
	mTBI	128.9 ± 12.7	180.7 ± 6.4	286.5 ± 12.9			
PDH	Sham	100.0 ± 22.1	120.0 ± 11.7	200.5 ± 21.1		<0.01	
	mTBI	102.2 ± 13.6	146.5 ± 25.5	222.8 ± 23.6			
Glut-1	Sham	100.0 ± 8.5	91.9 ± 5.7	79.1 ± 2.5		<0.05	
	mTBI	94.4 ± 4.9	84.1 ± 4.0	83.7 ± 6.4			
Glut-3	Sham	100.0 ± 5.4	111.9 ± 8.8	105.8 ± 5.2			
	mTBI	103.1 ± 5.9	91.4 ± 3.3	110.0 ± 7.2			
MCT-1	Sham	100.0 ± 3.3	95.6 ± 4.3	93.2 ± 2.4			
	mTBI	103.7 ± 3.3	100.8 ± 2.7	97.9 ± 3.1			
MCT-2	Sham	100.0 ± 9.6	116.1 ± 10.3	144.6 ± 3.5		<0.01	
	mTBI	109.1 ± 10.1	96.9 ± 5.5	150.0 ± 8.9			
HK2	Sham	100.0 ± 5.6	112.5 ± 6.0	82.4 ± 4.6		<0.01	
	mTBI	103.8 ± 7.3	102.3 ± 5.2	82.8 ± 5.6			
GPR 81	Sham	100.0 ± 9.5	117.0 ± 13.6	97.3 ± 12.5			
	mTBI	111.4 ± 17.1	103.6 ± 10.7	98.0 ± 8.2			

*Data are presented as % change of expression level of the cerebellum in the sham group measured at week 10 post mTBI*.

*Data are mean ± SE (n = 9–10/group)*.

*P Value: Obtained from Two-Way ANOVA analysis. If P > 0.05, the value is not listed on the table*.

#### Expression in Hippocampus (Table 2, and Supplemental File 1)

The hippocampus plays a major role in learning and memory (36, 37). Aging process has decreased the expressions of majority gene measured. These genes include HK1, PFK, Glut-3, MCT-2 in contralateral hippocampus, PK, PDH, Glut-1, MCT-1 in both contralateral and ipsilateral hippocampus. Of note, the levels for HK1, PFK, and Glut-3 in ipsilateral hippocampus were lower at the 10th week and increased to similar levels as the same genes' levels in contralateral hippocampus at the 28th week. Only MCT-2 remained lower level in the ipsilateral hippocampus.

For HK2 and GPR81, indicators of abnormal brain glucose utilization, aging had no effect for HK2 mRNA in the contralateral hippocampus, and for GPR81 mRNA in both contralateral and ipsilateral hippocampus. There was an age-related increase in HK2 mRNA in ipsilateral hippocampus only (*P* < 0.05).

In regard to the TBI effect, expression of two mRNAs, PK, and PDH, were significantly lower compared with that in the sham group in both contralateral and ipsilateral hippocampus. In contrast, the expression of both HK2 and GPR81, indicators of abnormal glucose metabolism, were increased in contralateral and ipsilateral hippocampus. mTBI did not affect PFK, Glu-1, Glut-3, MCT-1, and MCT-2 mRNA levels in either the contralateral and ipsilateral hippocampus. For HK1 expression in the ipsilateral hippocampus, the interaction of time X treatment was significant (*P* < 0.05). Thus, we compared the sham and mTBI groups at each individual time point. mTBI decreased hippocampal HK1 expression only at the 10th week post mTBI (*P* < 0.05).

#### Expression in Cortex (Table 3 and Supplemental File 2)

Similar to the hippocampus, aging also decreased expressions for some genes important for glucose transport and metabolism in cortex (Table 3). These genes include HK1, PDH in the contralateral cortex and Glut-3, MCT-2 in both the contralateral and ipsilateral cortex. In contrast to these decreases, the expression of PK was increased, and Glut-1 exhibited a U shape change with aging in the contralateral cortex. Also, in contrast to the hippocampus, expressions for HK2 and GPR81 were significantly decreased with aging in both the contralateral and ipsilateral cortex.

In regard to the mTBI effect, although the ipsilateral cortex was the directly injured site, we did not detect significant mTBI effects on the genes measured in either the contralateral or the ipsilateral cortex. There were three exceptions: lower mRNA of PFK at week 19 in contralateral cortex, and lower mRNAs of MCT-1 and HK2 at week 28 in the ipsilateral cortex for mTBI mice. Because of significant interactions of mTBI X time (*P* < 0.05), mTBI effects were examined at each individual time point, rather than an as overall main effect that covers all three times points, for PFK in the contralateral cortex and MCT-2 and HK2 in the ipsilateral cortex.

Thus, for cortical expressions of the above genes, the aging-related effect was still significant as observed in the hippocampus, but the main mTBI effect was not significant except when mTBI and Time interactions were significant, and mTBI effect was measured at individual time points.

#### Expression in Hypothalamus (Table 4 and Supplemental File 3)

Aging caused reversed U shape change for HK1, PFK, Glut-3, and MCT-2; and decreased expressions for PK and HK2 in the hypothalamus. Regarding the mTBI effect, there were no significant mTBI effects on expressions of gene measured in the hypothalamus, except that HK2 mRNA was higher in mTBI groups.

#### Expression in Brainstem (Table 5 and Supplemental File 4)

Aging elicited decreases in expression of PK, PDH, Glut-1, Glut-3, MCT-1, MCT-2 in the brainstem. Regarding the mTBI effect, there were no significant mTBI effects on the expressions of genes important for glucose metabolism in the hypothalamus.

As for the HK2 and GPR81, although aging caused decreases in their expressions, mTBI increased their expressions in the brainstem.

#### Expression in Cerebellum (Table 6 and Supplemental File 5)

Aging caused increases in the expression of PK, PDH, MCT-2 and decreases in Glu-1and HK2 in the cerebellum. mTBI did not significantly affect cerebellar gene expression except for an increase in PK expression.

In summary, the following mTBI effects were observed during the chronic phase of experimental mTBI: (1) decreased expression of PK and PDH only in the hippocampus; (2) increased expression of HK2 in the hippocampus, hypothalamus, and brainstem; (3) increased expression of GPR81 in the hippocampus and brainstem. Additionally, aging significantly affected the expression of the majority of genes in all brain regions and the patterns of alterations were gene and brain region specific.

## Discussion

Closed head concussive mTBI models have gained more attention in recent years as it “result in greater heterogeneity, … may more accurately depict human injury, particularly in the mild end of the spectrum”(38), especially for repetitive concussive brain injuries. On the other hand, there are more confounding factors that could influence the injury severity and outcome of repetitive concussive mTBI model (39, 40). For example, the number and interval of repeats, the impact position, direction force and depth are all varied in similar models, plus the neurocognitive outcome were also tested by different methods and their methods setting. Thus, it is hard for direct comparison of our model with other similar mTBI models. We continue to enhance our understanding and characterization of other aspects of the mTBI model we used in the current study.

Regardless, we found the observed neurocognition impairment neurodegeneration in the corpus callosum in our model to be in general agreement with findings from other similar models (41, 42), and prolongation of certain neurobehavioral impairments, such as spatial learning and memory similar to other concussive brain injured models (39, 40, 42–44). Of note, these mice exhibited normal NSS scores consistent with those in the sham group, possibly because NSS scores are more likely to change during the acute phase of TBI (33, 34).

Silver staining revealed that the most dramatic histologic damage occurred in the corpus callosum (CC). The corpus callosum is the largest white matter tract in the brain and is very sensitive to brain damage (41, 45, 46). It contains bundles of axons vital to information processing; and transmits motor, sensory and cognitive information across the hemispheres. Thus, neurodegeneration in the corpus callosum is consistent with the detected neurobehavioral alterations in mTBI mice, ands suggest that the model is suitable for study of the chronic phase effects of mTBI.

We previously reported that hippocampal and cortical expression of genes important for brain glucose utilization were all altered by TBI from 6 h to 28 days post brain injury (35). Those observed temporal alterations in gene expression corresponded closely to temporal alterations in brain glucose utilization reported in TBI patients and experimental animals during the acute and subacute phases of TBI (12–17, 47). The focus of the current study was the long-term impact of mTBI on expression of the same set of genes.

In comparison with our previous results in the acute phase of TBI induced by controlled cortical impact (35), the expression of genes encoding glucose or lactose transporters was not affected by mTBI in any of the brain regions measured. Further, expression of genes encoding HK1 and PFK, two of four rate limiting enzymes in glucose metabolism, was not significantly altered in any of the assessed brain regions. In contrast, expression of genes that encode two other key enzymes in glucose metabolism, PK and PDH, were significantly decreased only in the hippocampus, but not in any other brain regions tested. An increased cerebellar expression of PK by mTBI was even noted. Thus, the decreased PK and PDH expressions in hippocampus are tissue specific. These results suggest that the hippocampus is the most vulnerable brain region exhibiting sustained effects of mTBI, and that the other brain regions might have recovered from acute injury. Although the current study observations do not allow for determination of why the hippocampus was more vulnerable to the effects of mTBI than were other brain regions, clinical observations have revealed that the hippocampus exhibits prolonged impairments in glucose metabolism when compared with other brain regions, as detected by PET (16).

Although cortical expression of genes important for brain glucose utilization was not significantly altered, cortical expression of HK2 and GPR81 was greater in the mTBI group than in the sham group. The same observation was evident in the brainstem, but not in the hippocampus and cerebellum. These findings suggest that the damage by second injury might be brain region and metabolic pathway specific.

The mice used in the current study were 4 months old when the multiple brain injuries (mTBI) were completed, and the study duration was 28-weeks or 6.5-months post mTBI. Thus, the ages of mice ranged from ~4 to ~10–11 months old, which are comparable to ages from ~25 to ~40 years in humans (48, 49), consistent with the aging process from young adults to middle aged adults. We found that the majority of gene encoding proteins important for glucose metabolism were decreased during this time period in most of the brain regions tested. These results are consistent with the decreased glucose utilization determined by positron emission tomography (PET) imaging in middle aged compared with young adults (50, 51).

A limitation of the current study is that only gene expression in different brain tissues was measured. Without knowing expression profiles in different type of cells in the same regions, our results can only give overall profiles of this set of gene expression in the whole brain regions tested. We suspect that the results might vary in different types of cells in the same brain regions. Further measurements at cell specific levels are needed because cell to cell communication in the same region can provide a more detailed understanding of how TBI impairs brain glucose utilization (52). Thus, use of more advanced technologies, such as signal cell gene expression measurements (53–55), will be needed for future investigations. Regardless, the current results establish a foundation that will inform further research. Also, our current results cannot demonstrate any causative relationship between decreased expression of PK and PDH with impaired neurocognitive performance in mTBI mice. We plan to use knock out and overexpression models to modulate these two genes in the hypothalamus and to further evaluate their role in brain glucose metabolism and impaired neurocognitive performance in mTBI mice.

In summary, we demonstrate herein a mouse model of chronic mTBI that exhibited persistent decreased hippocampal expression of genes important in brain glucose homeostasis, in association with memory impairments, for up to 6 months post mTBI. Additionally, tissues from different brain regions responded differently during the chronic phase of mTBI. These data provide novel information regarding the long-term effects of brain injury on brain glucose utilization, neurobehavioral recovery, and their inter-relationships.

## Data Availability Statement

The raw data supporting the conclusions of this article will be made available by the authors, without undue reservation.

## Ethics Statement

The animal study was reviewed and approved by Washington DC VA Medical Center's Animal Care and Use, Research Safety and Research and Development Committees.

## Author Contributions

JZ conceived the experiments and wrote the first draft of the manuscript as PI of the project. LH and JZ performed the experiments and the statistical analyses. JZ, MPB, and MRB designed the experiments. JZ, MPB, DT, and MRB contributed to interpretation of data. JZ and MRB revised the manuscript. All authors contributed to and have approved the final manuscript.

## Conflict of Interest

The authors declare that the research was conducted in the absence of any commercial or financial relationships that could be construed as a potential conflict of interest.

## Supplementary Material

Supplemental File 1 Gene expression in hippocampus.

Supplemental File 2 Gene expression in cortex.

Supplemental File 3 Gene expression in hypothalamus.

Supplemental File 4 Gene expression in brainstem.

Supplemental File 5 Gene expression in cerebellum.
