# Supplementary material for: Chronic Neurobehavioral Impairments and Decreased Hippocampal Expression of Genes Important for Brain Glucose Utilization in a Mouse Model of Mild TBI
Source: Front Endocrinol (Lausanne). 2020 Sep 18;11:556380. doi: 10.3389/fendo.2020.556380 (PMC7531511; doi:10.3389/fendo.2020.556380)
Supplement: Supplementary file 1 [file Presentation_1.PPTX]

## Slide 1
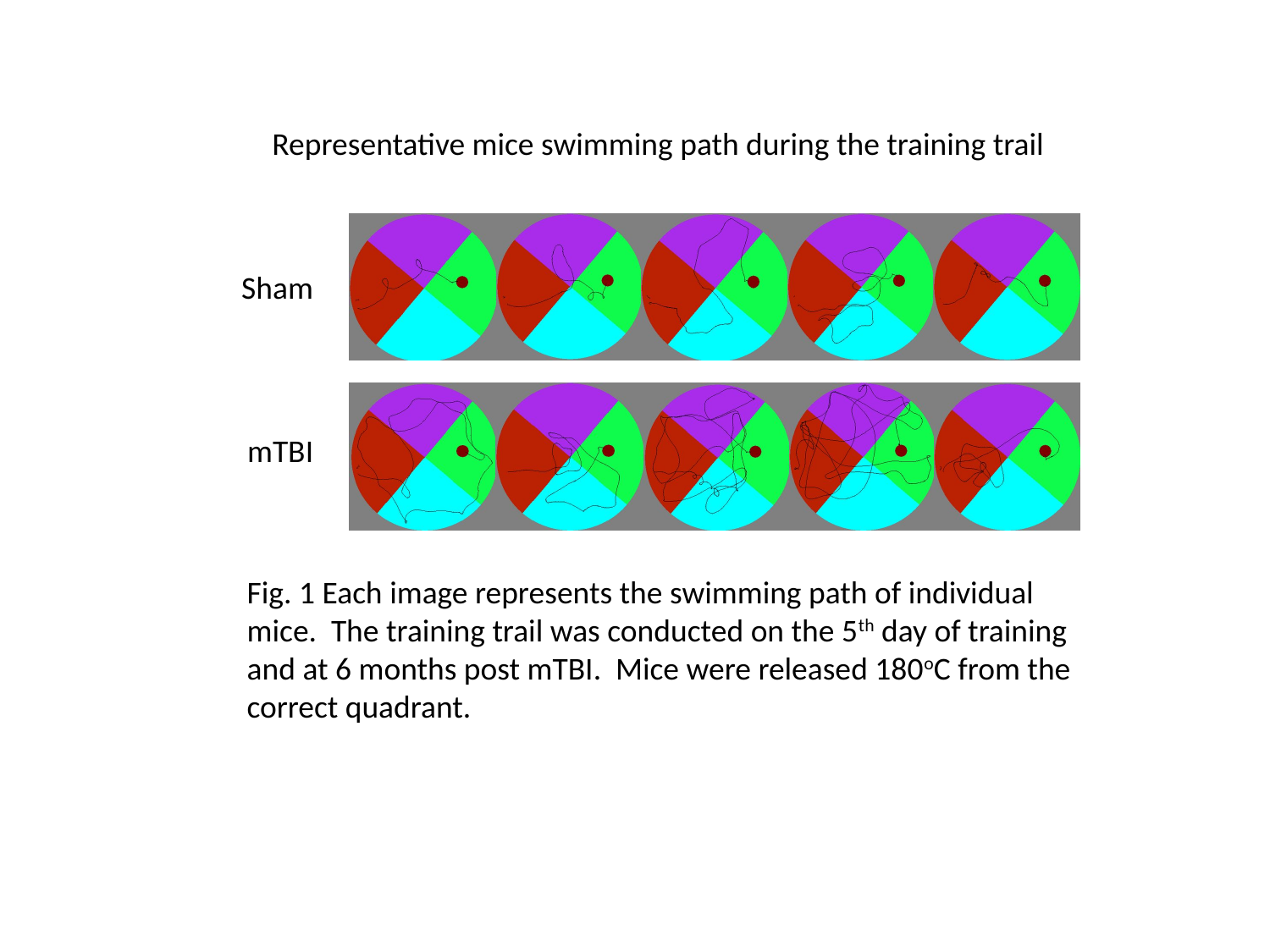

Representative mice swimming path during the training trail
Sham
mTBI
Fig. 1 Each image represents the swimming path of individual mice. The training trail was conducted on the 5th day of training and at 6 months post mTBI. Mice were released 180oC from the correct quadrant.

## Slide 2
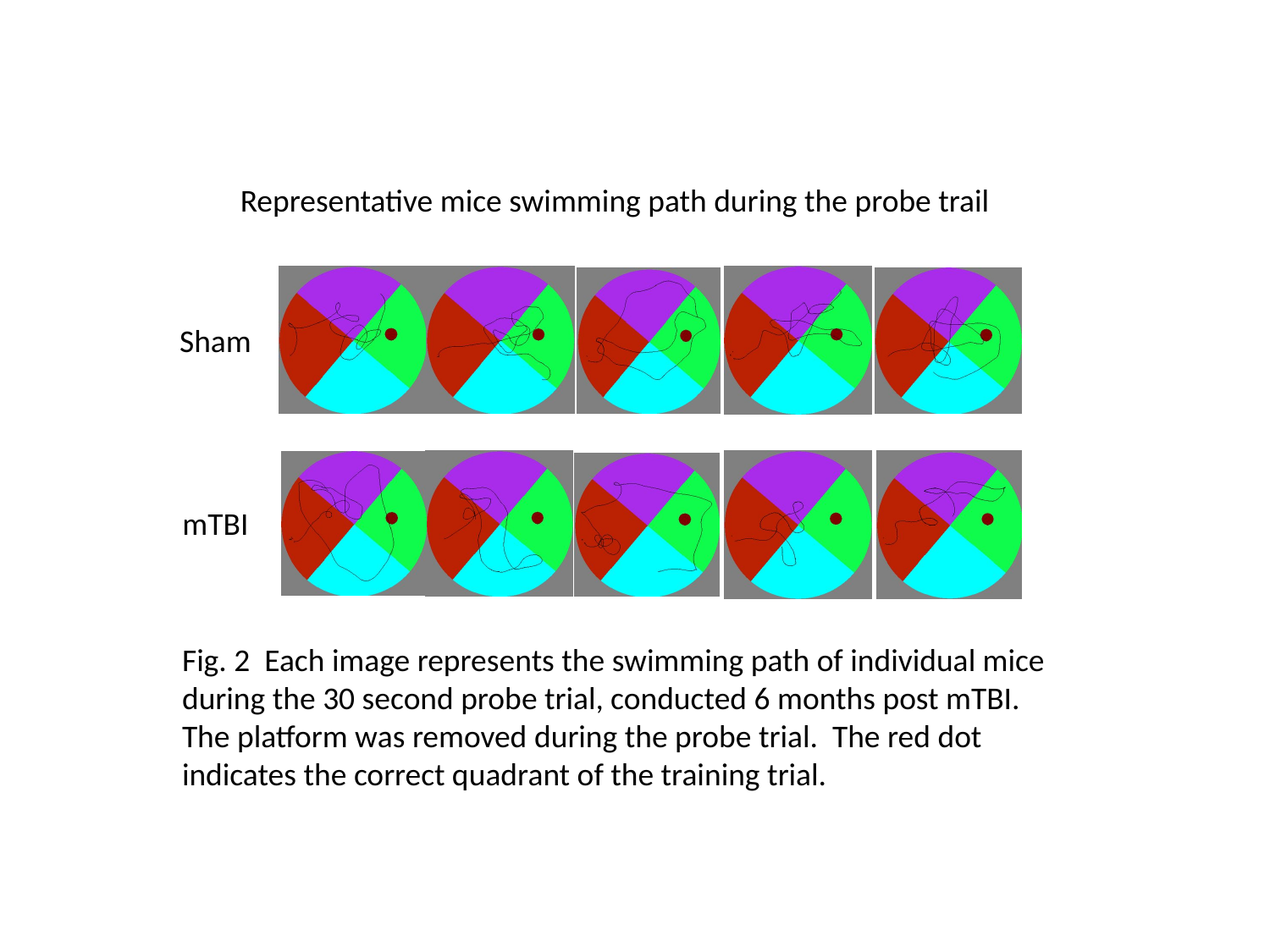

Representative mice swimming path during the probe trail
Sham
mTBI
Fig. 2 Each image represents the swimming path of individual mice during the 30 second probe trial, conducted 6 months post mTBI. The platform was removed during the probe trial. The red dot indicates the correct quadrant of the training trial.

## Slide 3
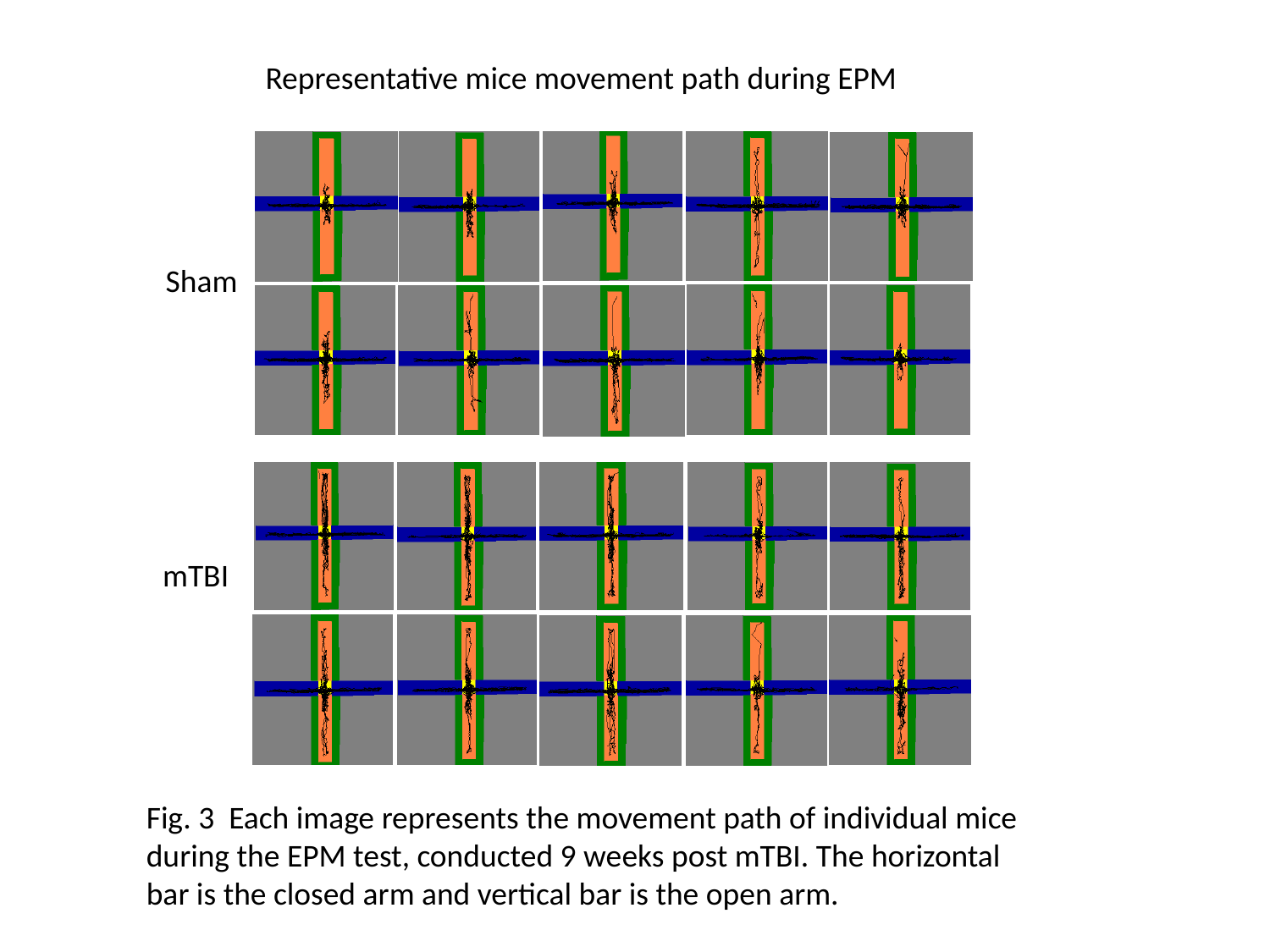

Representative mice movement path during EPM
Sham
mTBI
Fig. 3 Each image represents the movement path of individual mice during the EPM test, conducted 9 weeks post mTBI. The horizontal bar is the closed arm and vertical bar is the open arm.
